# Impact of Telehealth on Pediatric Direct Primary Care: Is Virtually Showing Up Valuable?

**DOI:** 10.7759/cureus.86122

**Published:** 2025-06-16

**Authors:** Austin Lee, Nicholas G Belt, Keili Mistovich, R Justin Mistovich, Andrew Hertz

**Affiliations:** 1 Orthopedic Surgery, Case Western Reserve University School of Medicine, Cleveland, USA; 2 Pediatrics, Zest Pediatrics, Cleveland, USA; 3 Orthopedic Surgery, MetroHealth Medical Center, Cleveland, USA

**Keywords:** community pediatrics, cost saving, direct primary care, healthcare finance, telehealth services

## Abstract

Objective: Direct primary care (DPC) is a transformative alternative to the fee-for-service insurance-based model, offering unlimited patient access for a monthly fee. With growing interest in DPC within pediatrics, our primary objectives were to evaluate how increased access through DPC influences multimodal telehealth utilization and assess its potential to reduce in-person care encounters and costs, including office, urgent care, and emergency department (ED) visits.

Methods: A cross-sectional electronic member survey of a multi-physician pediatric DPC network was distributed in April 2023, which assessed the utilization and impact of telehealth modalities and their ability to avoid in-person healthcare visits. Data from the chargemaster of a nearby children’s hospital were used as a proxy for the mean costs of pediatric office, urgent care, and ED visits to calculate cost savings for members.

Results: There were 59 parent responses out of 155 for a response rate of 38%; 100% (59/59) reported texting questions to their doctor, 93% sent pictures to the doctor, 88% directly telephoned the doctor, and 22% used video chat. The minimum mean healthcare expenditure avoided by utilizing telehealth modalities is $1,171.13 per year per family. Patient satisfaction with the DPC model demonstrated a Net Promoter Score (NPS) of 98/100.

Conclusion: Our study demonstrates that utilization of telehealth modalities in DPC reduces the frequency of in-person office, urgent care, and ED visits and leads to family cost savings. These findings offer valuable insight for healthcare agencies and professional societies developing policies to support pediatric DPC as a sustainable model that delivers value to families, employers, and payers alike. Limitations include regional cost variations and unspecified total avoided visits, and the number of pediatric patients per family. This may result in an underestimation of family savings. Further research should include a larger sample size with clear specifications on the number of visits avoided and the number of pediatric patients treated.

## Introduction

Direct primary care (DPC) is an evolution of traditional fee-for-service medical practices that focus on personalized, direct relationships between doctors and families [1]. DPC eliminates barriers to care commonly encountered in the conventional healthcare system, such as limited access, time constraints, and high patient volumes, which often hinder high-quality care [2, 3]. Rather than dealing with insurance requirements and potentially cumbersome restraints, DPC practices operate on a subscription membership basis in which families pay a monthly fee directly to the doctor’s office [4]. This subscription covers all traditional office-based costs without the additional burden of copays and insurance-related fees, thereby liberating doctors to devote their time to practicing evidence-based medicine while spending less time on billing and administrative tasks. Recently, interest in the DPC model has been growing among pediatricians [5].

Telehealth modalities (e.g., text messaging, video calls, and phone calls) are fundamental to a pediatric DPC model. They provide families with quick access directly to their pediatrician for many straightforward clinical questions. These methods not only enhance convenience and accessibility but also foster more collaborative relationships between pediatricians and families. By focusing on building traditional, longitudinal medical relationships and leveraging modern telehealth capabilities, the DPC model aims to improve patient satisfaction, reduce healthcare costs, and deliver the highest quality pediatric care. This study aims to evaluate the role of telehealth within a pediatric DPC network by (1) characterizing the utilization of various telehealth modalities, (2) estimating the cost savings associated with avoided in-person visits, and (3) assessing overall patient and family satisfaction with a pediatric DPC model that embraces telehealth modalities. By addressing these objectives, we aim to clarify the clinical and economic value of telehealth integration in pediatric DPC. 

## Materials and methods

Patient satisfaction survey

We performed an electronic member survey within a multi-physician pediatric DPC practice network across three office locations in northeast Ohio. Three questions were asked to assess the utilization and impact of telehealth modalities within the DPC model and the ability of these modalities to avoid in-person office visits, urgent care visits, or emergency department (ED) visits. The first question identified the methods by which patients communicated with their pediatrician: utilizing a Health Insurance Portability and Accountability Act (HIPAA)-compliant texting service to send texts to the doctor’s cell phone, sending a clinical picture to the doctor’s cell phone, direct phone call with the doctor, or a video chat with the doctor. The second question specified which further in-person services were avoided through this telehealth model: in-person doctor’s office visit, after-hours or weekend urgent care visit, or ED visit. Patients were not limited to one answer. The last question was used to generate a Net Promoter Score (NPS), a standardized metric measuring client loyalty and satisfaction widely used in both industry and healthcare [6]. NPS is a calculation based on the simple question, “How likely is it that you would recommend our business/service to a friend or colleague?” on a scale from 0 (not likely) to 10 (very likely). A score of nine to 10 is considered a promoter, seven to eight is considered passive, and a score of one to six is considered a detractor [7]. To calculate the score, the percentage of detractors is subtracted from the percentage of promoters. The score can range from -100 to 100, and a score of greater than 0 indicates that the percentage of promoters outweighs the percentage of detractors.

Practice-enrolled parents were sent an electronic survey link via SurveyMonkey (Symphony Technology Group, Menlo Park, CA) through email at the beginning of April 2023, with one reminder later that month. All families within the network were included in the survey. Respondents were divided into two cohorts based on DPC membership duration (< six months vs. > six months) to explore whether length of enrollment influenced telehealth utilization or perceived value. This stratification was chosen a priori to evaluate potential early versus established user experience. However, no formal statistical subgroup analyses were performed.

Cost analysis

We also sought to understand one component of the fiscal value of a DPC membership and included telehealth benefits through an analysis of avoided healthcare expenditures. To estimate costs for in-person healthcare visits potentially avoided through telehealth, we used the publicly available charge master from Nationwide Children’s Hospital as a baseline for visit costs. Specifically, we referenced the hospital’s discounted cash prices for commonly billed visit types, including pediatric office visits (Current Procedural Terminology (CPT) codes 99213 and 99214), urgent care visits (CPT codes 99203 and 99204), and emergency department visits (CPT codes 99283 and 99284). These values served as standardized cost estimates to quantify potential family savings associated with telehealth utilization in the DPC model. [8]. We chose this hospital because it was the closest facility to the locations within the DPC network with a fully downloadable chargemaster containing all relevant charges. Levels of evaluation and management service (levels 1, 2, 3, 4, or 5) are categorized based on the complexity of the visit, the extent of history taken, the examination performed, and the degree of medical decision-making involved [9]. To account for the difference in pricing based on differing levels of evaluation and management, we consulted with institutional physicians and administrative personnel to determine a reasonable assumption that 70% of avoided visits would have been classified as level 3 evaluation and care (CPT codes 99213, 99203, 99283), and 30% would have been classified as level 4 (CPT codes 99214, 99204, 99284). The cost for level 3 evaluation and care visits was pediatric office ($252), urgent care ($306), and ED ($720). The cost for level 4 evaluation and care visits was pediatric office ($351), urgent care ($459), and ED ($1,080). The avoided visit rates from the survey were multiplied by the respective CPT code costs to estimate potential savings. The mean healthcare expenditure avoided by utilizing telehealth modalities was calculated by summing the savings from avoided pediatric office visits, urgent care visits, and ED visits.

## Results

Patient satisfaction survey

The survey was sent to a total of 155 families that were members of the DPC network. There was a total of 59 responses for a response rate of 38%. In total, out of the 59 families who responded, there were high rates of telehealth utilization: 100% (59/59) reported texting questions to their doctor’s cell phone, 93% (55/59) sent clinical photos to the doctor’s cell phone, 88% (52/59) placed a direct telephone call to the doctor, and 22% (13/59) used video chat (Table 1). These communication modalities prevented at least one doctor’s office visit for 98% (58/59) of members, an after-hours or weekend urgent care visit for 92% (54/59) of members, and an ED visit for 69% (40/59) of members. Patient satisfaction with the DPC model demonstrated an NPS of 98.

**Table 1:** Survey questions and results

Questions	< 6 months	> 6 months	Total
Which of the following methods of communication have you used to reach your pediatrician (select all that apply)?	% Yes	% Yes	% Yes
Text the doctor	100% (16/16)	100% (43/43)	100% (59/59)
Send a picture to the doctor's cell phone	88% (14/16)	95% (41/43)	93% (55/59)
Phone call with the doctor	69% (11/16)	95% (41/43)	88% (52/59)
Video chat with the doctor	13% (2/16)	26% (11/43)	22% (13/59)
Has your ability to text, send a picture, video chat, or have a phone call directly with your doctor allowed you to avoid needing to take your child in for an in-person consultation?	% Yes	% Yes	% Yes
Doctor's office visit	94% (15/16)	100% (43/43)	98% (58/59)
After-hours or weekend urgent care visit	88% (14/16)	93% (40/43)	92% (54/59)
Emergency department visit	53% (8/16)	74% (32/43)	69% (40/59)
How likely is it that you would recommend our practice to a friend or colleague?			
Net Promotor Score	94	100	98

Of those 59 responses, 16 (27%) were considered new members and 43 (73%) were considered established members. In the cohort of established patients (who had been members for six months or more), 100% (43/43) reported having texted questions directly to the pediatrician’s cell phone, 95% (41/43) sent images to the pediatrician’s cell phone, 95% (41/43) placed a direct phone call to the pediatrician, and 26% (11/43) reported using video chat for consultation, indicating a high utilization rate of the telehealth features of the DPC model. Importantly, 100% of this cohort (43/43) reported avoiding an in-person doctor’s office, 93% (40/43) avoided an after-hours or weekend urgent care visit, and 74% (32/43) avoided an ED visit at least once (Table 1).

For families who had been members for less than six months, telehealth utilization patterns remained high; 100% (16/16) reported texting, 88% (14/16) sent photos, 69% (11/16) placed a telephone call, and 13% (2/16) had a video chat, indicating early adoption of these communication modalities. This also allowed these patients to avoid a doctor’s office visit (94%, 15/16), after-hours or weekend urgent care visit (88%, 14/16), and ED visit (50%, 8/16) at least once.

Cost analysis

The mean healthcare expenditure avoided by utilizing telehealth modalities was calculated to be $1,171.13 annually per family (Table 2). These estimated savings were compared to the mean annual membership cost per patient ($1,440 determined from internal membership records), indicating that this care modality significantly defrayed the costs of annual membership.

**Table 2:** Calculation of avoided healthcare costs CPT: Current Procedural Terminology

CPT code	Cost	Percent avoided	Average cost avoided	CPT code distribution
99213	$252	98%	$246.96	70%
99203	$306	92%	$281.52	
99283	$720	69%	$496.80	
Subtotal			$1,025.28	$717.70
99214	$351	98%	$343.98	30%
99204	$459	92%	$422.28	
99284	$1,080	69%	$745.20	
Subtotal			$1,511.46	$453.44
Total estimated avoided cost				$1,171.13

## Discussion

Although the DPC model is relatively new, there has been significant growth in recent years, with an estimated 1,400 such practices across the United States in 2021 [2]. This study contributes to the limited body of research on DPC models, especially pertaining to the pediatric patient population. Importantly, this study characterizes the role and value of telehealth services within this model. We aimed to first assess the utilization of the offered telehealth services and, second, the financial benefits of this feature. Additional analysis demonstrated the overall patient experience with the DPC model. Our data found high utilization rates of telehealth services, with families frequently using text messaging (100%), sending pictures (93%), making direct phone calls (88%), and conducting video calls (22%) with their pediatricians. These telehealth modalities thereby allowed families to reduce unnecessary in-person visits, including pediatric office visits (95%), urgent care visits (92%), and ED visits (69%). This use of the telehealth modalities within the DPC model ultimately resulted in a substantial average annual cost savings of $1,171 per patient against an annual average membership cost of $1,440. These savings, which are likely conservative, offset nearly the entire amount of membership. This analysis highlights the financial benefits of the DPC model within pediatric healthcare, demonstrating considerable cost savings through telehealth use. While we suspect the value of membership overall is greater than this single area of savings, we believe this information is useful to healthcare agencies and professional medical societies weighing how to best develop policies supporting the benefits and value of pediatric DPC membership for families, employers, and payers. 

It is important to note that these survey findings and financial calculations were performed without full knowledge of the number of specific appointments avoided or the total number of children cared for per family. Therefore, our data essentially describes the benefits families receive from utilizing the telehealth modalities and DPC model at least once. It is reasonable to assume that families with multiple children and/or multiple reasons for medical visits would see far greater cost savings.

In addition to the clear financial benefits, the added convenience of avoiding in-person visits due to these telehealth modalities further adds to the value of the DPC model and likely the high NPS. NPS is widely used in industry, with a positive score being well-regarded and any score above 50 suggesting good performance [10]. Within healthcare, studies have shown that NPS is a valid tool to assess patient experience and identify patient populations for whom the experience can be improved [11, 12]. This performance on the validated, patient-reported NPS metric illustrates the quantifiable success of direct primary care, including the fact that telehealth modalities utilized in this manner do not detract from, and instead likely enhance, patient and family experiences.

These findings align with previous studies indicating that DPC can lead to significant cost savings and improved patient outcomes. For instance, DPC has been shown to reduce emergency room visits and hospital admissions, leading to lower overall healthcare costs [2, 13]. The high NPS in our study also aligns with other reports of high patient satisfaction in DPC practices [14-16].

While this study provides significant insight into the benefits of telehealth within a DPC model, there were several limitations. First, the value for the mean healthcare expenditure avoided by utilizing telehealth modalities is likely an underestimate. The calculation assumed that each family only avoided one of each category of visits (pediatric office, urgent care, and ED) per year per child. In reality, families may have avoided multiple visits in each category. Additionally, facility fees typically associated with in-person visits were not accounted for, and some health systems are now charging for email correspondence, potentially increasing the cost savings further. Second, the cost analysis used the discounted cash prices from the Nationwide Children’s Hospital charge master, which may not accurately reflect regional cost variations. Third, survey data relied on self-reported responses. These may be subject to recall bias or overestimation of avoided visits. Fourth, the survey response rate might indicate that families more engaged and satisfied with services were more likely to respond. Furthermore, the survey did not collect demographic or practice-level data (e.g., number of children, patient age, parental education, or insurance status), which may have influenced telehealth use and cost savings and limited subgroup analysis. Lastly, the study included a relatively small sample size of 59 families within a single DPC network. Thus, these findings may not be generalizable to other populations or healthcare settings.

## Conclusions

Telehealth communication modalities are widely utilized in DPC, reducing the need for in-person office, urgent care, and ED visits. Our study characterizes the ways in which patients use these modalities, as well as identifies the benefits that patients receive from telehealth utilization. Despite study limitations, our data demonstrate clear net healthcare cost savings. Additionally, telehealth modalities provide the added convenience of avoiding in-person visits, further benefiting families utilizing these services. Future studies should incorporate larger sample sizes and multi-site designs to validate and expand upon these exploratory findings. Furthermore, they should include a clear specification of the number of specific visits avoided as well as the number of pediatric patients treated in totality. 
